# Transport of Ca^2+^ and Ca^2+^-Dependent Permeability Transition in Rat Liver Mitochondria under the Streptozotocin-Induced Type I Diabetes

**DOI:** 10.3390/cells8091014

**Published:** 2019-08-30

**Authors:** Konstantin N. Belosludtsev, Eugeny Yu. Talanov, Vlada S. Starinets, Alexey V. Agafonov, Mikhail V. Dubinin, Natalia V. Belosludtseva

**Affiliations:** 1 Laboratory of mitochondrial transport, Institute of Theoretical and Experimental Biophysics, Russian Academy of Sciences, Institutskaya 3, Pushchino, 142290 Moscow Region, Russia; 2 Department of Biochemistry, Cell Biology and Microbiology, Mari State University, pl. Lenina 1, Yoshkar-Ola, 424001 Mari El, Russia

**Keywords:** diabetes mellitus, mitochondria, calcium, Ca^2+^ uniporter, MPT pore, lipid pore, palmitic acid

## Abstract

Although diabetes mellitus is known to be a disease associated with mitochondrial dysfunction, not everything is clear about mitochondrial Ca^2+^ transport and Ca^2+^-induced permeability transition in diabetic cells. The objective of this work was to study the operation of MCU and Ca^2+^-dependent mitochondrial permeabilization in the liver cells of Sprague-Dawley rats under the streptozotocin-induced type I diabetes. It was shown that two weeks after the induction of diabetes, the rate of Ca^2+^ uptake by the mitochondria of diabetic animals increased ~1.4-fold. The expression of MCU and MICU1 subunits did not change, yet the quantity of dominant-negative MCUb channel subunits was almost twice as lower. The organelles also became more resistant to the induction of CsA-sensitive MPT pore and less resistant to the induction of CsA-insensitive palmitate/Ca^2+^-induced pore. The mitochondria of diabetic liver cells also showed changes in the lipid matrix of their membranes. The content of fatty acids in the membranes grew, and microviscosity of the lipid bilayer (assessed with laurdan) increased. At the same time, lipid peroxidation (assessed by the production of malonic dialdehyde) was stimulated. The paper discusses the consequences of the diabetes-related changes in mitochondria in the context of cell physiology.

## 1. Introduction

Diabetes mellitus (DM) is one of the most common metabolic diseases which is associated with either impairment of insulin secretion (type I diabetes) or tolerance of cells to insulin (type II diabetes). The common pathological syndrome in both cases is an increased level of blood glucose (hyperglycemia), which, in the long run, inflicts serious damage to many organs and systems of the organism. At the intracellular level, one of the main targets of diabetes are mitochondria. There are a lot of diabetes-related disorders, developing in the structure and function of mitochondria of various tissues and organs: Impairment of mitochondrial biogenesis, remodeling of the mitochondrial cell network, disorders in the system of oxidative phosphorylation, hyperproduction of reactive oxygen species, etc. [1,2,3].

One of the manifestations of mitochondrial dysfunction in diabetes mellitus are disorders in the mitochondrial Ca^2+^ ion transport systems. As a major buffer system of this ion, mitochondria are involved in the regulation of intracellular Ca^2+^ homeostasis and, in addition, Ca^2+^ activates a number of mitochondrial respiratory enzymes and enzymes of the Krebs cycle. On the other hand, excessive accumulation of Ca^2+^ in mitochondria can lead to the opening of mitochondrial pores and initiation of cell death [4,5].

Ca^2+^ enters mitochondria via Ca^2+^ uniporter, a protein complex consisting of the channel subunit MCU, its paralog (the dominant-negative channel subunit MCUb), the gate subunits MICU1-2, and the subunits EMRE and MCUR1 [4,5]. It is generally accepted that the regulation of Ca^2+^ uniporter is mediated by the interaction of MCU with MCUb and MICU1 and by changes in the ratio of these proteins in the complex. For example, overexpression of MCUb subunit impairs the ion-transporting function of the Ca^2+^ uniporter [6]. A low level of MICU1 expression in the heart, as compared to the liver, explains why heart mitochondria have a low activation threshold for Ca^2+^ transport and a lower Ca^2+^ capacity [7].

As mentioned above, excessive accumulation of Ca^2+^ in mitochondria leads to the opening of Ca^2+^-dependent mitochondrial pores, which is a key step in the mechanism of the programmed cell death. The mitochondrial permeability transition pore (MPT pore) is considered to be a protein mega-channel, which includes proteins of the inner and outer membrane of mitochondria. It is not exactly clear which proteins form the pore—yet currently, they are believed to be either the mitochondrial ATP synthase or adenylate translocator. It is also established that MPT pore includes cyclophilin D, a regulatory protein targeted by the pore inhibitor cyclosporin A (CsA) [5,8].

Earlier we showed that, in addition to the CsA-sensitive MPT pore, there was another variety of pores which could be opened in mitochondria in the presence of saturated fatty acids and Ca^2+^. Those pores were not responsive to CsA and other modulators of the CsA-sensitive MPT pore. The studies performed on artificial lipid membranes (BLM and liposomes) showed that the mechanism of membrane permeabilization in that case was based on the ability of saturated fatty acid anions to form tight complexes with Ca^2+^ in the lipid bilayer followed by their segregation into solid-crystalline membrane domains. The process of phase separation was shown to be accompanied by the formation of hydrophilic lipid pores [5,9].

The studies on the mitochondrial Ca^2+^ transport and MPT pores in the diabetic cells (at both types of diabetes) have been carried out for a long time, yet the data obtained are rather contradictory. In different studies of type I diabetes in animals, both the uptake of Ca^2+^ by cells and mitochondria and the opening of MPT pore have been reported to be either stimulated or repressed [10,11,12,13,14]. Of particular interest are the data that type-I diabetic liver mitochondria are more resistant to the opening of MPT pores [15]. The mechanisms of these effects are often not clear, although they should presumably depend on the species and age of the animal, as well as the type of tissue.

In this work, we have examined changes in the systems of mitochondrial Ca^2+^ transport (Ca^2+^ uniporter) and MPT pore opening in the liver of rats with streptozotocin-induced type-I diabetes. Taking into account the fact that diabetes also causes disorders in lipid metabolism, we have further investigated the formation of palmitate/Ca^2+^-induced pore in liver mitochondria of diabetic animals. The results have shown that: (1) streptozotocin-induced type-I diabetes increases the rate of Ca^2+^ uptake by liver mitochondria of Sprague-Dawley rats, which may be associated with a decreased expression of MCUb; (2) under streptozotocin-induced diabetes, the Ca^2+^ capacity of rat liver mitochondria is enhanced, and the amplitude of CsA-sensitive Ca^2+^/P_i_-induced mitochondrial swelling is lowered; and (3) treatment of Sprague-Dawley rats with streptozotocin decreases the resistance of their liver mitochondria to palmitate/Ca^2+^-dependent CsA-insensitive permeability transition.

## 2. Materials and Methods

### 2.1. Animals

The laboratory animals (Sprague-Dawley male rats) were treated in accordance with the European Convention for the Protection of Vertebrates used for experimental and other purposes (Strasbourg, 1986) and the principles of the Helsinki Declaration (2000). All the protocols were approved by the Institute of Theoretical and Experimental Biophysics RAS Ethics Committee (Order No. 173/k of 03.10.2011, Protocol No. 04/2019 of 05.03.2019).

### 2.2. Induction of Diabetes

Experiments were performed on Sprague-Dawley male rats with induced diabetes mellitus (*n* = 5) and without diabetes (*n* = 5). Diabetes was induced by a single injection of streptozotocin (STZ; 70 mg/kg, IP). This is considered to be the optimal diabetogenic dose: At higher concentrations, STZ can have a toxic effect not only on the pancreatic β-cells but on the cells of liver and kidney as well [16,17]. Control rats received an equal volume of vehicle (0.1 M citrate buffer, pH 4.5). Rats with blood glucose over 300 mg/dL were considered diabetic. Animals were sacrificed after two weeks, while blood glucose (BG) and body weight (BW) were regularly monitored.

### 2.3. Isolation of Rat Liver Mitochondria

Mitochondria were isolated from the liver of Sprague-Dawley rats (150–200 g) by differential centrifugation as described earlier [18]. The homogenization buffer contained 210 mM mannitol, 70 mM sucrose, 1 mM EDTA, and 10 mM Hepes/KOH buffer, pH 7.4. Subsequent centrifugations were performed in the same buffer, except that, instead of EDTA, 100 μM EGTA was used. Final suspensions contained 70–80 mg of mitochondrial protein/mL, as determined by the Lowry method [19].

### 2.4. Ca^2+^ Uptake by Mitochondria

The concentration of Ca^2+^ in the reaction medium (external [Ca^2+^]) was measured with an ion-selective electrode [20]. The reaction medium contained 150 mM sucrose, 50 mM KCl, 2 mM KH_2_PO_4_, 5 mM succinate, 1 μM rotenone, 5 μM EGTA, and 10 mM Hepes/KOH buffer, pH 7.4. The measurements were carried out in in a stirred cuvette at room temperature (~22 °C). The concentration of mitochondrial protein was 1–1.5 mg/mL. In the experiments, 25 μM Ca^2+^ was added to the reaction medium every 60 s. After several additions, external [Ca^2+^] increased, indicating a massive release of the ion from the organelles due to the opening of MPT pore in the inner mitochondrial membrane. The amount of Ca^2+^ released upon permeability transition (defined as Ca^2+^ capacity) was used as a measure of the MPT pore opening probability.

### 2.5. Mitochondrial Respiration and Ca^2+^/O

The rate of oxygen consumption was measured polarographically with a Clark-type gold electrode Oxygraph-2k (O2k, OROBOROS Instruments, Innsbruck, Austria) at 25 °C under continuous stirring [20]. The reaction medium contained 130 mM KCl, 5 mM KH_2_PO_4_, 5 mM succinate, 1 μM rotenone, 10 µM EGTA, and 10 mM Hepes/KOH, pH 7.4. The concentration of mitochondrial protein was ~0.5 mg/mL. The respiratory control of succinate-oxidizing mitochondria was in the range of four to five. The Ca^2+^/O ratio was estimated under limited conditions of Ca^2+^ load, by measuring stimulated respiration after the addition of 200 nmoles of CaC1_2_ to the suspension of respiring mitochondria [21].

### 2.6. Mitochondrial Swelling

The swelling of mitochondria (0.4 mg/mL) was measured as a decrease in absorbance at 540 nm (*A*_540_) in a stirred cuvette at room temperature (~22 °C) using a USB-2000 spectroscopy fiber-optic system (Ocean Optics, Dunedin, FL, USA) [20]. The incubation medium contained 210 mM mannitol, 70 mM sucrose, 5 mM succinate, 10 μM EGTA, 1 μM rotenone, and 10 mM Hepes/KOH buffer, pH 7.4. The rate of swelling (*V*_max_ = Δ*A*_540_/min per mg protein) was calculated as a change in absorbance within the first 30 s from the beginning of the high-amplitude swelling. The amplitude of swelling (%) was determined 3 min after the beginning of high-amplitude swelling. The change in absorbance upon the addition of alamethicin (7.5 μg/mL) was taken as 100% swelling.

### 2.7. Fluidity of the Mitochondrial Membrane

Fluidity of the mitochondrial membrane was determined by measuring the fluorescence intensity of laurdan (6-dodecanoyl-2-dimethylaminonaphthalene) in temperature-controlled (37 °C) 96-well plates [22]. Freshly prepared liver mitochondria were suspended (0.5 mg/mL) in a medium containing 210 mM mannitol, 70 mM sucrose, 5 mM succinate, 10 μM EGTA, 1 μM rotenone, 1 mM KH_2_PO_4_, and 10 mM Hepes/KOH buffer, pH 7.4. After that, 1 μM laurdan was added, and the samples were incubated in dark for 30 min at 37 °C. Laurdan fluorescence was excited at 340 (20) nm, and the emission was measured at two wavelengths: 430 (20) and 495 (10) nm—using a Tecan Spark 10 M reader (Tecan, Männedorf, Switzerland). The generalized polarization (GP) values were calculated as follows: GP = (I_430_ − I_490_)/(I_430_ + I_495_), where I_430_ and I_495_ were the laurdan emission intensities at the respective wavelengths. GP can theoretically assume values from −1 (the least ordered state) to +1 (the most ordered state).

### 2.8. Lipid Peroxidation

Lipid peroxidation in the suspension of liver mitochondria was estimated spectrophotometrically by measuring the levels of thiobarbituric acid-reactive substances (TBARS). The TBARS assay quantifies the levels of malondialdehyde (MDA) and other minor aldehyde species through their reaction with thiobarbituric acid. The concentration of TBARS was calculated using the molar absorption coefficient of the colored TBA–MDA complex (E_535_ = 1.56 × 10^5^ M^−1^·cm^−1^) [23].

### 2.9. Electrophoresis and Immunoblotting of Mitochondrial Proteins

To prepare samples for quantifying the levels of mitochondrial proteins, aliquots of native mitochondria (2 mg/mL) were solubilized in Laemmli buffer in Eppendorf tubes and heated for 3 min at 95 °C. Aliquots of the mitochondrial samples equalized by the protein concentration (10 µg of mitochondrial protein) were applied to the gel lanes and subjected to electrophoresis followed by Western blot analysis. Mitochondrial samples were separated by 12.5% SDS-PAGE and transferred to a 0.45 µm nitrocellulose membrane (Amersham, Germany). The proteins of PageRuler Prestained Protein Ladder (Thermo Fisher Scientific, Waltham, MA, USA) was used as markers. After overnight blocking, the membrane was incubated with the appropriate primary antibody. The monoclonal rabbit anti-MCU (#14997), anti-CBARA/MICU1 antibody (#12524) and anti-ANT2/SLC25A5 (#14671) antibodies were from Cell Signalling technology Inc (Danvers, MA, USA). The total OXPHOS Rodent WB Antibody Cocktail (ab110413), containing α-subunit of complex V (CV-ATP5A-55 kDa), anti-VDAC1 (ab154856) and the polyclonal rabbit antibodies Anti-CCDC109B (ab170715), anti-cyclophilin F (CypD) (ab64935) and anti-ANT1 (ab102032) were from Abcam (Cambridge, GB). The immunoreactivity was detected using the appropriate secondary antibody conjugated to horseradish peroxidase (#7074, Cell Signaling technology Inc., Danvers, MA, USA). Peroxidase activity was detected with ECL chemiluminescence reagents (Pierce, Rockford, IL, USA). The relative levels of the detected proteins were visualized using an LI-COR system (LI-COR, Lincoln, NE, USA). Optical density measurements were performed using LI-COR Image Studio software.

### 2.10. Fatty Acid Composition of Mitochondrial Phospholipids

The fatty acid composition of mitochondrial phospholipids was determined using the method of gas chromatography. Lipids were extracted from liver mitochondria using the modified method by Bligh and Dyer [24]. Mitochondria (1 mg/mL) were placed in a centrifuge tube, then 200 μL of water and 900 μL of chloroform/methanol mixture (2:1, *v*/*v*) were added. The mixture was kept at room temperature for 30 min and stirred periodically. Heptadecanoic acid (C17:0, 15 μg/mL) in hexane was added to each sample as an internal standard. Chloroform and water/methanol layers were separated by centrifugation at 10,000× *g* for 10 min at 4 °C. The lower layer of the extract containing the lipid fraction was collected and evaporated to dryness in a stream of argon at 25 °C. The obtained fractions were methylated with one volume of 5% sulfuric acid/methanol (*v*/*v*) at 100 °C for 3 h. The reaction was stopped by the addition of three volumes of 5% K_2_CO_3_ (*v*/*v*). Methyl ethers were extracted by four volumes of hexane and then evaporated in a stream of nitrogen to a volume of 100 μL. Samples were analyzed using a Chromatek-Crystal 5000 gas chromatography system (Chromatek, Yoshkar-Ola, Russia). On the basis of the chromatograms obtained, the following indexes were calculated: TFA, the total amount of fatty acids; SFA, the total amount of saturated fatty acids; PUFA, the total amount of polyunsaturated fatty acids; and the unsaturation index (UI), which was calculated by multiplying the amount of a fatty acid by the number of double bonds in its structure.

### 2.11. Statistical Analysis

The data were analyzed using the GraphPad Prism 7 (GraphPad Software, San Diego, CA, USA) and Microsoft Excel 2016 (Microsoft, Redmond, WA, USA) software and were presented as means ± SEM of four to five experiments. Statistical differences between the means were determined by a two-tailed *t*-test; *p* < 0.05 was considered to be statistically significant.

## 3. Results

Table 1 shows data on the level of blood glucose and weight of control and experimental rats. One can see that initially, the animals were approximately of the same weight. However, animals from the experimental group, which were injected with streptozotocin (70 mg/kg), gained much less weight than control animals over the experimental two-week period. By the end of this period, the level of blood glucose in experimental animals was significantly higher than in animals of the control group.

### 3.1. The Development of Diabetes Stimulates Ca^2+^ Uptake by Rat Liver Mitochondria

**Figure 1:**
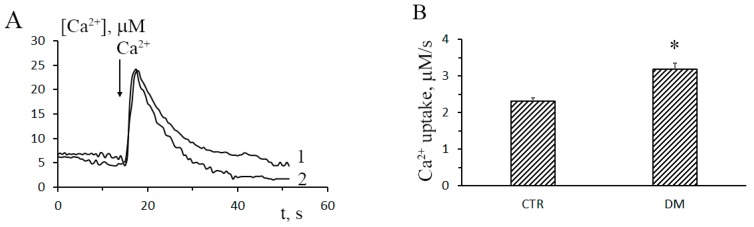

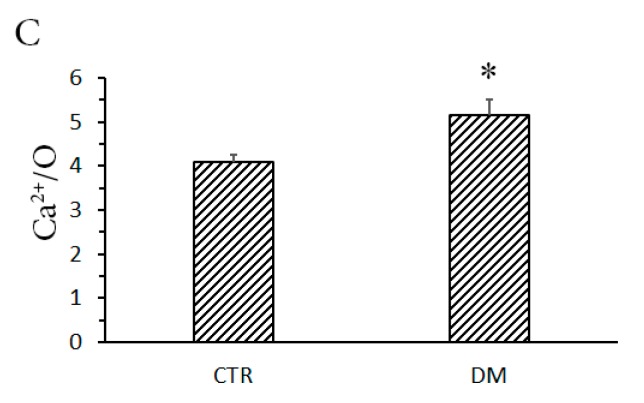
Diabetes leads to increased rates of Ca^2+^ uptake by rat liver mitochondria. (**A**) The dynamics of Ca^2+^ uptake (25 μM) by liver mitochondria of control (trace 1) and STZ-treated (trace 2) rats. (**B**) Rates of Ca^2+^ uptake by liver mitochondria of control and STZ-treated rats. The incubation medium contained 150 mM sucrose, 50 mM KCl, 2 mM KH_2_PO_4_, 5 mM succinic acid, 5 µM EGTA, 1 µM rotenone, and 10 mM Hepes-KOH, pH 7.4. The concentration of mitochondrial protein in the cuvette was 1 mg/mL. (**C**) The Ca^2+^/O ratio of liver mitochondria of control and STZ-treated rats. The incubation medium contained 120 mM KCl, 5 mM NaH_2_PO_4_, 5 mM succinic acid, 5 µM EGTA, 1 µM rotenone, 1 µM cyclosporin A (CsA), and 10 mM HEPES-KOH, pH 7.4. Mitochondrial oxygen consumption was stimulated by the addition of 200 μM CaCl_2_. The concentration of mitochondrial protein in the cuvette was ~0.5 mg/mL. Values are given as means ± SEM (*n* = 5). * *p* < 0.05 compared to controls.

In this work, we have analyzed structural-functional features of the system of mitochondrial Ca^2+^ transport in the liver of animals with type-1 STZ-induced DM. Figure 1A shows the dynamics of Ca^2+^ uptake by liver mitochondria of control and STZ-treated rats (curves 1 and 2, respectively). Upon the induction of DM, the rate of Ca^2+^ uptake by rat liver mitochondria increased approximately 1.4-fold. The mitochondrial Ca^2+^/O ratio of diabetic animals was 1.2-fold of that of control animals (Figure 1C). Thus, one could conclude that liver mitochondria of diabetic animals accumulated Ca^2+^ faster and more efficiently, as compared to the control organelles.

The influx of Ca^2+^ into mitochondria is mediated by Ca^2+^ uniporter, a complex of proteins of the inner mitochondrial membrane, which includes channel subunits (MCU and MCUb), regulatory subunits (MICU1-2, EMRE, and MCUR1), and a number of other proteins. It is believed that transport of Ca^2+^ through the uniporter is regulated by the ratio between the channel subunits MCU and MCUb and the regulatory subunit MICU1 [4,5,6,7]. Since liver mitochondria of diabetic animals accumulate Ca^2+^ faster comparatively to the control organelles, one can assume that diabetes changes the relative content of these subunits in the mitochondrial membrane.

**Figure 2:**
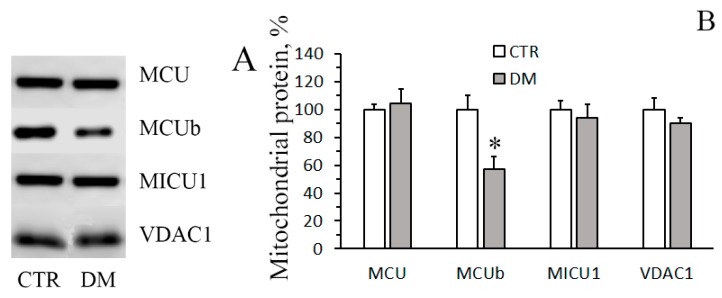
Contents and ratios of Ca^2+^ uniporter proteins in the liver mitochondria of control (CTR) and diabetic (DM) rats. (**A**) Western blot analysis of members of the Ca^2+^ uniporter protein family (MCU, MCUb, and MICU1). (**B**) Summarized data of densitometric band analysis of these proteins. In these experiments, VDAC1 was used as a marker. Values are given as means ± SEM (*n* = 4). * *p* < 0.05 compared to controls.

Figure 2A shows immunoblots of liver mitochondrial Ca^2+^ uniporter proteins of control and STZ-treated rats. It can be seen that the content of MCU and MICU1 almost did not change, whereas the content of MCUb (dominant-negative channel subunit of the uniporter) decreased almost by half. Correspondingly, the MCU/MCUb ratio grew from 3.0 ± 0.7 in control animals to 6.7 ± 0.8 in STZ-treated rats (Figure 2B).

On the basis of the data obtained, one can conclude that the increase in the rate of Ca^2+^ uptake by liver mitochondria of diabetic animas is underlain by a decrease in the relative content of the dominant-negative subunit of Ca^2+^ uniporter MCUb.

### 3.2. The Development of Diabetes Increases the Resistance of Liver Mitochondria to the Opening of MPT Pore

**Figure 3:**
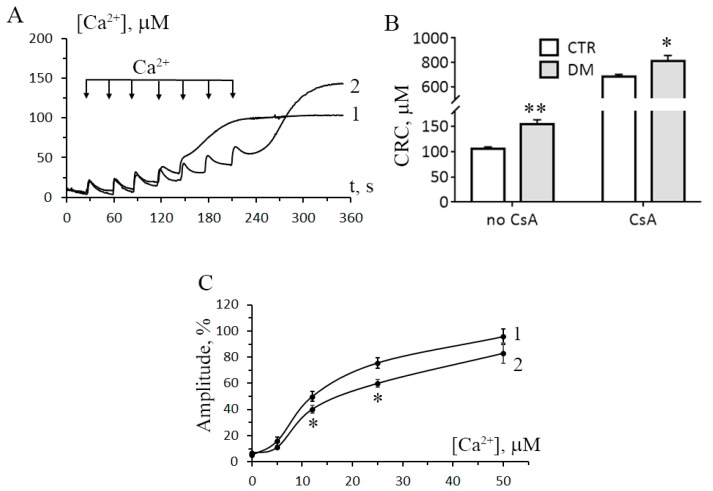
Diabetes increases the resistance of rat liver mitochondria to the opening of mitochondrial permeability transition (MPT) pore. (**A**) Changes in the external [Ca^2+^] upon successive addition of small Ca^2+^ doses (25 μM) to the suspension of liver mitochondria of control (trace 1) and STZ-treated (trace 2) rats. (**B**) Ca^2+^ capacity of liver mitochondrial of control and diabetic animals in the absence and presence 1 μM CsA. The values are given as means ± SEM (*n* = 5). The medium composition was as indicated in Figure 1A. (**C**) The amplitude of Ca^2+^-induced swelling of liver mitochondria of control (curve 1) and diabetic (curve 2) rats versus Ca^2+^ concentration. The incubation medium contained 210 mM mannitol, 70 mM sucrose, 5 mM succinate, 10 μM EGTA, 1 μM rotenone, and 10 mM Hepes/KOH buffer, pH 7.4. The concentration of mitochondrial protein was 0.4 mg/mL. The values are given as means ± SEM (*n* = 4). * *p* < 0.05 compared to controls; ** *p* < 0.01 compared to controls.

The next objective of our work was to examine how the development of diabetes would affect the opening of MPT pore in liver mitochondria. We monitored two parameters associated with the MPT pore opening: Ca^2+^ capacity of mitochondria and Ca^2+^-induced swelling of the organelles. The mitochondrial Ca^2+^ capacity is related to the threshold concentration of Ca^2+^, necessary for the pore to open. One of the ways to assess this parameter is to introduce Ca^2+^ into the suspension of mitochondria in small successive doses, and the number of such additions until MPT is triggered will reflect the Ca^2+^ capacity of the organelles. Figure 3A shows the results of such experiments. It can be seen that for mitochondria of STZ-treated rats, the number of successive Ca^2+^ additions (and, therefore, the threshold pore-opening Ca^2+^ concentration) was greater comparatively to the control. The Ca^2+^ capacity of diabetic mitochondria increased 1.5-fold. This implies that their resistance to the opening of MPT pore would also be higher. A similar conclusion can be drawn from the data on the Ca^2+^/P_i_-dependent swelling of rat liver mitochondria. As seen in Figure 3C, the amplitude of Ca^2+^-dependent swelling of diabetic mitochondria was lower than the amplitude of swelling of control organelles. It should be noted that in the presence of CsA, the Ca^2+^ capacity of mitochondria of diabetic animals was higher than that of control ones, with the difference being statistically significant.

The structure of MPT pore—which is believed to be a mega-channel penetrating both the inner and outer mitochondrial membranes—has yet to be determined. By now, adenylate translocator, ATP synthase, and cyclophilin D are considered to be essential components of the pore apparatus [8,25]. In the present work, we have examined how the levels of these proteins change when DM is induced.

**Figure 4:**
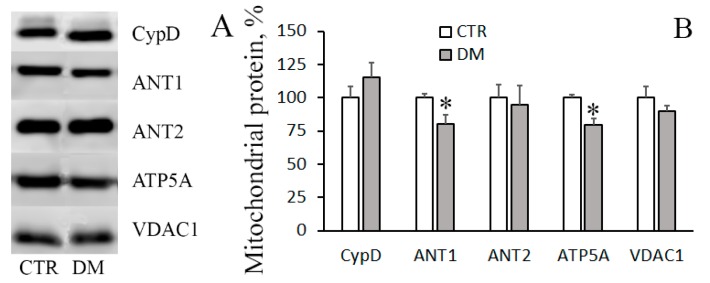
The levels of MPT-related proteins in the liver mitochondria of CTR and DM rats. (**A**) Western blot analysis of MPT-related proteins: CypD, ANT1, ANT2, and ATP5A. (**B**) Summarized data on the levels of these proteins in mitochondria. VDAC1 was used as a marker. Values are given as means ± SEM (*n* = 4). * *p* < 0.05 compared to controls.

Figure 4 shows immunoblots of cyclophilin D, adenylate translocator isoforms (ANT1 and ANT2), and α-subunit of ATP synthase of mitochondria isolated from liver of control and STZ-treated rats. One can see that the amount of cyclophilin D and ANT2 did not change with the development of DM. The levels of ANT1 and mitochondrial ATP synthase, on the other hand, were reduced, with the reduction being statistically significant. The decrease in the levels of MPT-related mitochondrial proteins may explain the enhanced resistance of the diabetic organelles to the opening of MPT pore.

### 3.3. The Development of Diabetes Decreases the Resistance of Mitochondria to the Formation of CsA-Insensitive Palmitate/Ca^2+^-Dependent Pores

Apart from CsA-sensitive MPT, mitochondria can undergo CsA-insensitive palmitate/Ca^2+^-dependent permeability transition [5]. The inductors of this type of permeability transition are saturated long-chain fatty acids—in particular, palmitic acid—contributing to the development of DM [26,27]. Our next step was to investigate how the development of DM would change the resistance of mitochondria to the induction of palmitate/Ca^2+^-dependent pores.

**Figure 5:**
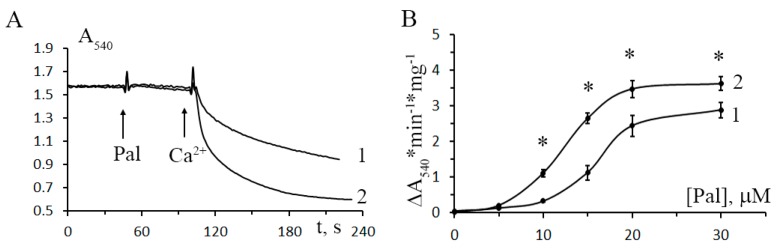
Effect of diabetes mellitus on the opening of palmitate/Ca^2+^-induced lipid pores in rat liver mitochondria. (**A**) Absorbance changes in the suspension of liver mitochondria of control (trace 1) and STZ-treated (trace 2) rats in response to the addition of 20 μM palmitic acid (Pal) and 30 μM Ca^2+^. (**B**) The rate of CsA-insensitive palmitate/Ca^2+^-induced swelling of liver mitochondria in control (curve 1) and diabetic (curve 2) rats versus palmitic acid concentration. The incubation medium contained 210 mM mannitol, 70 mM sucrose, 5 mM succinate, 10 μM EGTA, 1 μM rotenone, 1 μM CsA, and 10 mM Hepes/KOH buffer, pH 7.4. Additions: 30 μM Ca^2+^. The concentration of mitochondrial protein was 0.4 mg/mL. Values are given as means ± SEM (*n* = 5). * *p* < 0.05 compared to controls.

Figure 5 shows the dynamics of mitochondrial swelling (monitored by absorbance) in the suspensions of liver mitochondria of control and STZ-treated rats after the addition of 20 μM palmitic acid and 30 μM Ca^2+^. It can be seen that in diabetic mitochondria, the amplitude of swelling is higher comparatively to the control. Figure 5B shows the rate of CsA-insensitive palmitate/Ca^2+^-dependent mitochondrial swelling versus the concentration of palmitic acid. As seen in the figure, the rate of palmitate/Ca^2+^-dependent swelling of diabetic mitochondria is higher over the entire range of palmitic acid concentration. Thus, while developing resistance to the induction of CsA-sensitive MPT, mitochondria of diabetic animals become more sensitive to the induction of CsA-insensitive palmitate/Ca^2+^-dependent pores.

The formation of palmitate/Ca^2+^-induced pores in mitochondria depends on the composition and properties of the lipid component of the membrane [5]. In this regard, we have investigated how DM affects these parameters.

**Figure 6:**
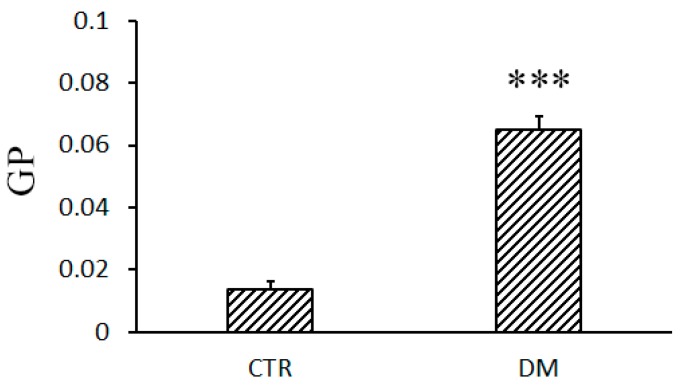
Fluidity of liver mitochondrial membranes of control and diabetic rats. The composition of the incubation medium was as indicated in Figure 5. Values are given means ± SEM (*n* = 5). *** *p* < 0.001 compared to controls.

Figure 6 shows changes in the fluidity of membranes of liver mitochondria of control and diabetic animals. One can see that in diabetic mitochondria, there is a statistically significant increase in the parameter GP calculated from the laurdan fluorescence data. This means that the mobility of water molecules in the region of lipid heads is lowered, which implies an increase in microviscosity of the mitochondrial membrane.

Table 2 shows data on the fatty acid composition of mitochondrial membranes (total pool of esterified and free fatty acids). It can be seen that the total amount of fatty acids in the mitochondrial membrane rises, with the content of polyunsaturated fatty acids growing most substantially (1.7-fold). Correspondingly, there is a statistically significant increase in the unsaturation index.

**Figure 7:**
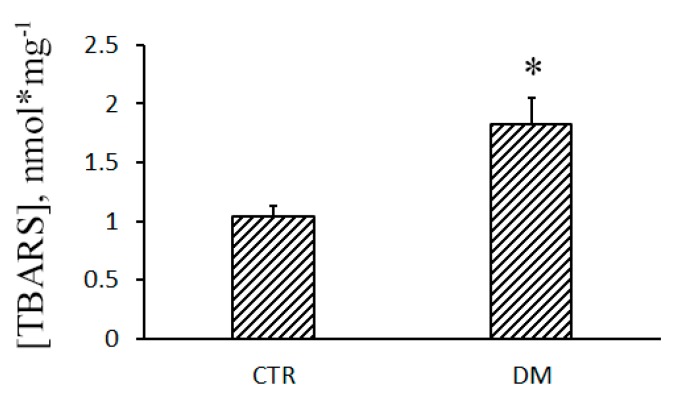
Diabetes stimulates lipid peroxidation in rat liver mitochondria. Lipid peroxidation was assessed by the level of thiobarbituric acid-reactive substances (TBARS) (MDA and other minor aldehyde species) in the liver mitochondria of control (CTR) and STZ-treated (DM) rats. Values are given as means ± SEM (*n* = 4). * *p* < 0.05 compared to controls.

In parallel with the increase in the content of polyunsaturated fatty acids in the mitochondrial membrane, lipids undergo an intense peroxidation. Figure 7 shows that the concentration of TBARS in diabetic mitochondria grows, which indicates enhanced lipid peroxidation and the development of oxidative stress.

## 4. Discussion

It is generally accepted that mitochondria are one of the main intracellular targets of DM. In diabetic cells, the mitochondrial network is damaged, the process of ATP synthesis is impaired, and oxidative stress is developed. Diabetes also affects Ca^2+^ homeostasis of the cell, which is regulated with a direct involvement of mitochondria. Disorders in the system of mitochondrial Ca^2+^ transport not only affect the bioenergetic parameters of the cell but can also induce cell death via the opening of mitochondrial pores. The purpose of our work was to study in more detail how type-I DM affects the system of Ca^2+^ transport, as well as the probability of pore formation, in rat liver mitochondria.

Studies on the effects of type-I DM on mitochondrial Ca^2+^ transport systems have been conducted for a long time. The uptake of Ca^2+^ by the organelles is primarily achieved through the mitochondrial Ca^2+^ uniporter complex, the basic component of which is the pore-forming protein MCU. The MCU pore is a highly selective Ca^2+^ channel, which transfers Ca^2+^ across the inner mitochondrial membrane and is associated with other subunits, both structural and regulatory: MCUb, MICU1-2, EMRE, and MCUR1 [4,5]. The literature data on the effect of type-I DM on mitochondrial Ca^2+^ transport is rather contradictory. It has been shown, for example, that the development of STZ-induced diabetes lowers the rate of Ca^2+^ uptake by mice heart mitochondria, and this effect has been revealed to be associated with the decreased expression of MCU [13,28]. In this study, we have found that the rate of Ca^2+^ uptake by liver mitochondria increased significantly in diabetic rats (1). Similar results on liver mitochondria were obtained earlier on the model of alloxan diabetes [12]. As shown here, the cause for the DM-related stimulation of Ca^2+^ uptake by rat liver mitochondria is not an elevated expression of MCU, but a decreased expression of the MCU paralogue, MCUb (Figure 2).

It is believed that the MCU pore is formed by 4 transmembrane channel subunits, which can be either MCU or MCUb. MCUb is a dominant-negative MCU paralog; its overexpression and presence in the uniporter complex impairs the Ca^2+^-transporting properties of the Ca^2+^ uniporter [6]. Owing to the reducing of the MCUb level, the ratio of MCU/MCUb in diabetic mitochondria grows more than 2-fold (Figure 2B), and this can explain the enhanced rates of mitochondrial Ca^2+^ uptake. At the same time, the expression of the gate subunit MICU1 in the liver of STZ-treated rats almost does not change. A similar observation was reported earlier for heart mitochondria [28]. It can, therefore, be assumed that the DM-related changes in the mitochondrial Ca^2+^ transport occurs primarily at the level of pore, rather than regulatory, subunits of the uniporter complex. Moreover, the structural-functional alterations in the Ca^2+^ uniporter complex seem to be tissue-dependent.

As mentioned above, excessive accumulation of Ca^2+^ in mitochondria leads to the opening of pores in the membrane of organelles: CsA-sensitive MPT pores and CsA-insensitive lipid palmitate/Ca^2+^-induced pores [5]. As a result, transmembrane ion gradients and membrane potential (Δψ_m_) collapse, and mitochondria swell, this leading to the rupture of their outer membrane and the release of proapoptotic proteins from the organelles. One of the factors facilitating the opening of MPT pores in mitochondria is oxidative stress, and our experiments show that the level of malondialdehyde (TBARS) in the liver mitochondria of STZ-treated rats grows (Figure 7), indicating an increased lipid peroxidation. Yet, surprisingly, these mitochondria become more resistant to the opening of MPT pores (Figure 3). Earlier, liver mitochondria of STZ-treated rats were shown to have an increased lag period before they start to swell due to the opening of MPT pores [15]. The increased resistance of the organelles to MPT might be underlain by metabolic changes, such as an increase in the content of coenzyme Q or cardiolipin [29]. In any case, this effect seems to depend on the type of tissue: in diabetic heart mitochondria, the probability of MPT pores to open increases [14].

To probe into the molecular mechanism of resistance of diabetic liver mitochondria to MPT, we assayed for levels of MPT-associated mitochondrial proteins: Cyclophilin D, ATP synthase, and adenylate translocator isoforms, ANT1 and ANT2 (Figure 4). It can be seen that in mitochondria of diabetic animals, the level of cyclophilin D remains almost unchanged. There is, however, a significant decrease in the level of ANT1 and ATP synthase—proteins that supposedly form the MPT pore channel. Thus, the increased resistance of diabetic mitochondrial to MPT may be underlain by a lowered expression of the MPT pore-forming proteins.

An important result of this work is the finding that liver mitochondria of diabetic rats become less resistant to the formation of CsA-insensitive palmitate/Ca^2+^-induced lipid pores (Figure 5). Diabetes is known to be accompanied by the impairment of lipid metabolism—and our results show that the content of total fatty acids in diabetic mitochondria grows substantially (Table 2). This can facilitate the formation of lipid pores in the mitochondrial membrane.

Formation of lipid pores in a membrane depends on the physicochemical properties and structure of its lipid bilayer. It was demonstrated that changes in the lipid composition and microviscosity of the membrane (induced by unsaturated cardiolipin or unsaturated free fatty acids) led to the stimulation of lipid pore formation—both in liposomes and mitochondria [18,30,31]. The results of this work show that the level of polyunsaturated fatty acids in the liver mitochondria of diabetic animals grows significantly (Table 2). Correspondingly, peroxidation of lipids goes up (Figure 7). The products of lipid peroxidation can alter membrane packing and increase its microviscosity [32]—exactly what we observed when diabetic mitochondria were probed with laurdan (Figure 6). Altogether, these data give an explanation for the increased lipid pore formation in the mitochondria of diabetic animals.

It should be noted that, as lipid pores, the palmitate/Ca^2+^-induced pores tend to close spontaneously, which can lead to the restoration of Δψ_m_. The restoration of Δψ_m_ was demonstrated in our previous work, in which we also suggested that these pores may be a nonspecific system for Ca^2+^ release from the organelles [33]. The formation of lipid pores can be considered as an emergency option for quick release of Ca^2+^ from mitochondria which, in contrast to the CsA-sensitive MPT pore, may not lead to the collapse of Δψ_m_ and damage of the organelles.

## 5. Conclusions

The results obtained in this work indicate that type-I diabetes mellitus leads to structural and functional rearrangements of the system responsible for the regulation of Ca^2+^ homeostasis in liver mitochondria. Liver is an organ responsible for detoxification of xenobiotics—and the one most affected by diabetes. It is important that mitochondria of liver cells adapt to the diabetes-related metabolic changes (the rate of Ca^2+^ uptake by the organelles grows, their sensitivity to MPT decreases, and the formation of short-lived lipid pores in the mitochondrial membrane is facilitated). These adaptive changes enhance the resistance of mitochondria to massive destruction, which would otherwise lead to cell death. At the same time, these changes seem to be tissue-specific: other tissues and organs can respond to the diabetic stress differently–in regard to both intensity and direction of the response.

## Figures and Tables

**Table 1:** Animal weights and biochemical characteristics in the studied groups.

	Control	DM
Initial BW, g	90.2 ± 6.6	86.5 ± 5.5
Final BW, g (after two weeks)	197.7 ± 3.6	135.1 ± 6.8 ****
BG (mg/dL)	125.9 ± 8.9	489.1 ± 16.5 ****

Values are given as mean ± SEM of the number of independent experiments indicated (*n* = 5). BG, blood glucose; BW, body weight. Initial weight of the animals was registered at the time of their injection with streptozotocin (STZ) or vehicle. **** *p* < 0.0001 compared to controls.

**Table 2:** DM-related changes in the fatty acid composition of the mitochondrial membrane (μg per mg of mitochondrial protein).

Fatty Acids	Control	DM1
C16:0	19.98 ± 1.05	25.98 ± 2.32 *
C18:0	15.07 ± 0.865	27.71 ± 2.72 **
C18:1	6.68 ± 0.24	10.49 ± 0.63 **
C18:2	13.99 ± 1.04	27.60 ± 3.59 *
C20:4	18.35 ± 1.35	26.71 ± 1.24 **
C24:1	0.71 ± 0.08	0.61 ± 0.05
C22:6	8.32 ± 0.84	12.18 ± 1.34
C24:0	0.53 ± 0.16	0.41 ± 0.12
TFA	83.01 ± 4.45	132.2 ± 14.77 *
SFA	35.31 ± 1.81	53.31 ± 5.67 *
PUFA	40.66 ± 2.76	69.44 ± 7.87 *
UI	158.3 ± 11.07	258.6 ± 28.92 *

The experimental conditions are described in Materials and Methods. TFA, total fatty acids; SFA, total saturated fatty acids; PUFA, total polyunsaturated fatty acids; UI, unsaturation index. * *p* < 0.05 compared to controls ** *p* < 0.01 compared to controls.
